# Focus on materials genome and informatics

**DOI:** 10.1080/14686996.2016.1246226

**Published:** 2017-01-06

**Authors:** Kiyoyuki Terakura, Ichiro Takeuchi

**Affiliations:** ^a^ National Institute for Materials Science, Tsukuba, Japan; ^b^ Materials Science and Engineering, University of Maryland, College Park, MD, USA

A key challenge in the field of materials sciences is the continued search for new materials with specific desired functions. There is currently no established, prescribed method for achieving this goal. Nevertheless, a variety of new materials with remarkable properties and functions have been discovered and synthesized in recent years, often as a result of lengthy, painstaking trials inspired by ideas from experienced researchers. This has stimulated this fundamental scientific field and led to numerous technological innovations. From the discovery of a new material up to its successful transfer to the industrial and commercial stages, there are two important intermediate steps in the process to consider.[1]


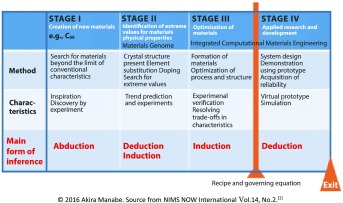


The discovery of a new material is generally followed by intensive efforts to optimize its properties and functions, occasionally complemented by attempts to further explore other potential functions of the material. Then, once the material is of sufficient quality, the transfer to technological applications begins.

There are therefore three stages of materials exploration, which can be described thus:

Stage I: Initial discovery of new materials

Stage II: Optimization of their properties and functions

Stage III: Optimization of technological processes for the preparation of a ready-to-use material with specific, desired functions

Naturally, the path to successful technological application is different for every material, and each stage presents its own challenges. The most significant overarching challenges for materials science are limitations in availability of energy and natural resources. To this end, the Materials Genome Initiative (MGI) was launched in 2011 in the USA. The MGI homepage states that its aim is ‘to help businesses discover, develop, and deploy new materials twice as fast’*.*[2] Inspired by MGI, several related projects have recently been launched worldwide. Materials informatics is a key component of the MGI and its associated initiatives. This is the materials science version of bio-informatics and cheminformatics, which is conceptually related to the ‘fourth paradigm’ (the dawn of data-intensive scientific discovery).[3]

Given the recent increased emphasis on ‘big data’ across multiple disciplines, it is fair to ask how materials informatics will fit into this new era of research. This is a somewhat controversial question. The largest existing databases based on experimental results from materials are about 5 × 10^5^ data elements in size,[4] and only part of this information is used in individual material analyses. Indeed, the combinatorial materials science community has been wrestling with this challenge for some time already.[5] Therefore, some people believe that materials science has not yet reached the big data stage. However, the rapid progress in computational science and microscopy techniques,[6] for example, is resulting in enormous amounts of output data. Perhaps materials science could now be on the verge of entering the big data era.

Finally, consideration should be given to the implications of materials informatics in materials research. There are two basic approaches in materials science. One is the deductive method, wherein the properties or functions of given materials are studied. The second method is inductive, whereby suitable materials are actively sought to satisfy desired properties or functions. This is commonly achieved by using scientists’ expertise and experience, and by mining existing or newly collected data. The latter is the so-called ‘materials design’ approach. The former is known as the direct problem and the latter the inverse problem. The inverse problem is generally much more difficult to solve than the direct problem, and therefore materials design is traditionally a challenging subject. We, as materials scientists, are hopeful that materials informatics may provide us with ways of solving inverse problems in future.

The articles in this Focus Issue of Science and Technology of Advanced Materials (http://www.tandfonline.com/stam) pay special attention to the practical aspects of materials informatics, the current situation of materials databases and the future of data mining in the field. Important keywords include database, data mining, machine learning, and artificial intelligence, to name but a few. Although the articles found herein will also be published individually, they will be later combined together to form a single special volume in both on the Web and in print. We hope this Focus Issue will contribute to the promotion of materials informatics as a new materials research approach, help researchers understand its current situation and future possibilities, and serve as a stepping stone for future advancement of this rich interdisciplinary field.
